# The application of ambroxol hydrochloride combined with fiberoptic bronchoscopy in elderly patients with severe pneumonia: A meta-analysis and systematic review

**DOI:** 10.1097/MD.0000000000028535

**Published:** 2022-01-28

**Authors:** Haowei Tang, Zhi Yuan, JingJie Li, Qun Wang, Weijie Fan

**Affiliations:** Department of Respiratory Medicine, Fenghua District People's Hospital, China.

**Keywords:** ambroxol hydrochloride, bronchoscopy, care, elder, pneumonia, review

## Abstract

**Background:**

The role of ambroxol hydrochloride combined with fiberoptic bronchoscopy in elderly patients with severe pneumonia remains unclear, we aimed to analyze this issue to provide evidences into the management of clinical pneumonia.

**Methods:**

We searched PubMed et al databases up to October 20, 2021 for the randomized controlled trials on the application of ambroxol hydrochloride combined with fiberoptic bronchoscopy in elderly patients with severe pneumonia. Related outcomes were extracted and analyzed. Review Manager 5.3 software was used for data analysis.

**Results:**

A total of 13 randomized controlled trials involving 1317 elderly patients (559 cases in the ambroxol hydrochloride + fiberoptic bronchoscopy group and 658 cases in the fiberoptic bronchoscopy group) with pneumonia were included. Meta-analyses indicated that the blood oxygen partial pressure [mean difference (MD) = 5.75, 95% confidence interval (CI) (3.80, 7.70)], blood oxygen saturation [MD = 6.43, 95% CI (4.39, 8.48)], oxygenation index [MD = 26.75, 95% CI (14.61, 38.89)] of experimental group was significantly higher than that of control group (all *P* < .001), the incidence of multiple organ failure [odds ratio = 0.42, 95% CI (0.31, 0.56), *P* < .001], mortality on day 28 [odds ratio = 0.44, 95% CI (0.33, 0.59)] of experimental group was significantly less than that of control group (all *P* < .001).

**Conclusions:**

The high-dose ambroxol hydrochloride combined with fiberoptic bronchoscopy is beneficial to improve the patient's blood gas indicators, and reduce mortality in elderly patients with severe pneumonia.

## 1 Introduction

Elderly patients with severe pneumonia have a lot of sputum, and are often unable to move freely, which can cause sputum to accumulate. If the sputum cannot be excreted in time, it will easily lead to aggravation of pneumonia and even multiple organ failure.^[1,2]^ According to relevant reports,^[3,4]^ the mortality of severe pneumonia varies from 50.27% to 71.06%. In clinical settings, patients were given conventional doses of ambroxol hydrochloride combined with vibration for expectoration on the bases of conventional treatment. This method has a certain effect, but the effect is not very satisfactory.^[5,6]^ How to effectively promote the expectoration to reduce the incidence and severity of pneumonia is one of the important concerns of current respiratory medicine.

Ambroxol hydrochloride is currently clinically mainly used as a common expectorant. Fiberoptic bronchoscopy has a high degree of visualization, which can absorb inflammatory secretions and sputum, and then lavage with normal saline to further facilitate the discharge of sputum.^[7]^ Therefore, in recent years, fiberoptic bronchoscopy has become a new method for the treatment of severe pneumonia. Research results^[8,9]^ show that the application of fiberoptic bronchoscopy irrigation treatment can effectively improve the clinical symptoms of patients with severe pneumonia. At the same time, studies^[10,11]^ have pointed out that ambroxol hydrochloride has a good function of dissolving sputum, but its sputum expectoration effect is not obvious. If combined with bronchoscopy for sputum suction, severe pneumonia may be effectively treated.^[12]^ At present, several studies^[13,14]^ have reported the use of high-dose and conventional-dose ambroxol hydrochloride in the treatment of severe pneumonia, the results remain controversial. Besides, due to the limitation of sample size, region, and research subjects, there is still a lack of systematic reviews on the superiority of those 2 treatments. Therefore, it is necessary to evaluate the relevant publications in this field through the method of meta-analysis. We aimed to analyze the effects and safety of high dosed ambroxol hydrochloride combined with fiberoptic bronchoscopy in elderly patients with severe pneumonia, to provide useful references for the treatment of clinical pneumonia.

## 2 Methods

This meta-analysis and systematic review was conducted and reported according to the Preferred Reporting Items for Systematic Reviews and Meta-Analyses (PRISMA).^[15]^

### 2.1 Search strategy

We searched 7 databases including PubMed, The Cochrane Library, Web of Science, CNKI, Wanfang Database, Weipu Database, and China Biomedical Document Service System. The search strategies used in this study were: (Mesh“ambroxol” OR Mesh“ambroxol hydrochloride” OR Mesh “Mucosolvan” OR Mesh “bronchoscope”) AND (Mesh “pulmonary infection” OR Mesh “pneumonia”). The search time limit was from the establishment of the database to October 20, 2021. For the search we used the combination of subject words and free words to expand the scope of search.

### 2.2 Inclusion and exclusion criteria

The inclusion criteria for this meta-analysis were: Study design: randomized controlled trial (RCT). Research objects and intervention measures: elderly patients aged >60 years, and the severe pneumonia was diagnosed according to relevant diagnostic criteria. The experimental group was given high-dose ambroxol hydrochloride (≥120 mg/d) combined with fiberoptic bronchoscope suction, and the control group was given conventional dose of ambroxol hydrochloride (≤90 mg/d) combined with vibration expectoration. The RCT reported related outcome indicators: including blood oxygen partial pressure, blood oxygen saturation, oxygenation index, incidence of multiple organ failure, and 28-day mortality.

The exclusion criteria for this meta-analysis were the research results were reported other than Chinese and English language. Studies in which the same results were published repeatedly. Studies with incomplete data or inaccessible data.

### 2.3 Quality assessment

The Cochrane Collaborations risk of bias tool^[16]^ was adopted by 2 authors independently to evaluate the methodological quality and risk of bias of the included RCTs. Any disagreements were resolved by discussion and consensus. This tool evaluated 7 specific domains including sequence generation, allocation concealment, blinding of participants and personnel, blinding of outcome assessment, incomplete outcome data, selective outcome reporting, and other issues. Each domain was rated as low risk of bias, high risk of bias, or unclear risk of bias according to the judgment criteria.

### 2.4 Statistical analysis

Review Manager 5.3 software (Cochran library, Australia) was used for data analysis. We performed a chi-square test to determine whether there was heterogeneity between the studies. The significance level was set to α = 0.05, and I^2^ was used to quantitatively analyze the heterogeneity. If *P* ≥ .1 or I^2^ ≤ 50%, the fixed effects model was used to analyze. Otherwise, the random effects model was used for analysis. The indicators of continuous data were represented by the mean difference (MD) and its 95% confidence interval (CI), and the odds ratio (OR) was used as the effect indicator for the binary variables. The publication bias of this study was qualitatively analyzed by the funnel plot and Egger test. The test level of this meta-analysis was set to α = 0.05.

## 3 Results

### 3.1 RCT selection and characteristics

**Figure 1:**
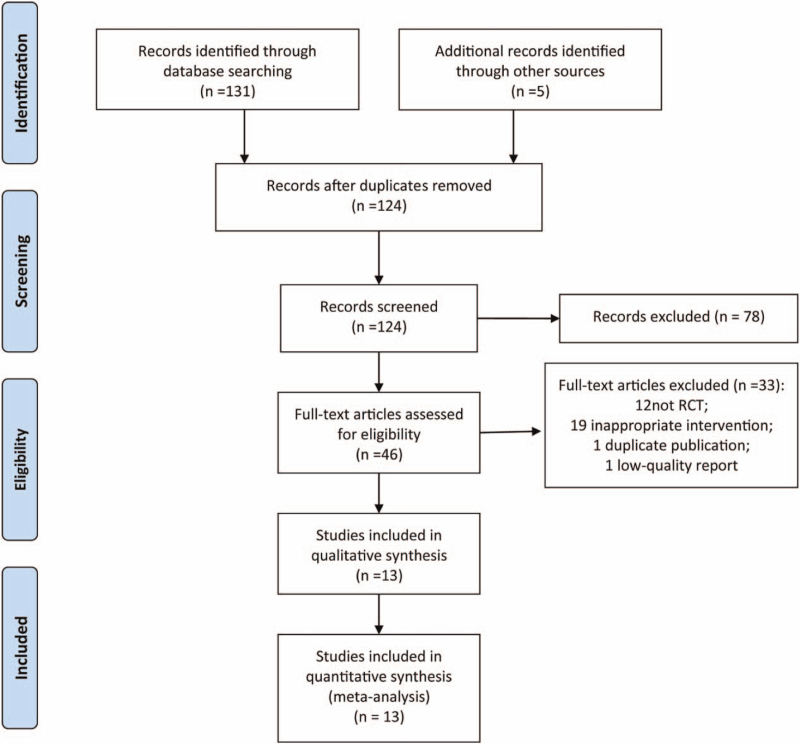
PRISMA flow diagram for study selection. PRISMA = Preferred Reporting Items for Systematic Reviews and Meta-Analyses.

A total of 13 RCTs^[17–30]^ were included in this study, and the literature selection process is shown in Figure 1. Among the included 13 RCTs, a total of 1317 elderly patients with pneumonia were included as research objects, including 559 cases in the experimental group and 658 cases in the control group. The basic information of each included RCT is shown in Table 1.

**Table 1:** The characteristics of include RCTs.

	Sample size			Age		Interventions	
Studies	Experimental group	Control group	Total	Experimental group	Control group	Experimental group	Control group
Li 2012	27	34	61	66.1 ± 11.2	63.0 ± 13.6	High-dose ambroxol hydrochloride (nebulized 120 mg/d for 5 days) combined with fiberoptic bronchoscope for sputum suction	Conventional dose (nebulized 90 mg/d for 5 days) combined with vibration expectoration instrument
Yao 2017	50	50	100	68.21 ± 8.26	68.55 ± 8.12	High-dose ambroxol hydrochloride (nebulized 1005 mg/d for 3 days) combined with fiberoptic bronchoscope for sputum suction	Conventional dose (nebulized 90 mg/d for 3 days) combined with vibration expectoration instrument
Niu 2017	35	34	69	73 ± 0.84	74 ± 0.95	High-dose ambroxol hydrochloride (nebulized 150 mg/d for 7 days) combined with fiberoptic bronchoscope for sputum suction	Conventional dose (nebulized 90 mg/d for 7 days) combined with vibration expectoration instrument
Su 2017	46	46	92	66.1 ± 4.6	66.7 ± 3.8	High-dose ambroxol hydrochloride (oral 30 mg/kg/day, for 5 days) combined with fiberoptic bronchoscope for sputum suction	Conventional dose (oral 60 mg/d for 5 days) combined with vibration expectoration instrument
Chen 2016	28	27	55	73.62 ± 7.89	73.15 ± 7.68	High-dose ambroxol hydrochloride (nebulized 600 mg/d for 7 days) combined with fiberoptic bronchoscope for sputum suction	Conventional dose (nebulized 60 mg/d for 7 days) combined with vibration expectoration instrument
Luo 2017	46	46	92	69.2 ± 3.7	68.4 ± 3.5	High-dose ambroxol hydrochloride (nebulized 600 mg/d for 3 days) combined with fiberoptic bronchoscope for sputum suction	Conventional dose (90 mg/d) combined with vibration expectoration instrument
Yu 2017	45	45	90	68.07 ± 3.39	67.49 ± 3.57	High-dose ambroxol hydrochloride (nebulized 120 mg/d for 5 days) combined with fiberoptic bronchoscope for sputum suction	Conventional dose (nebulized 60 mg/d for 5 days) combined with vibration expectoration instrument
Zeng 2014	144	140	284	77.9 ± 8.9	79.3 ± 7.5	High-dose ambroxol hydrochloride (nebulized 120 mg/d for 7 days) combined with fiberoptic bronchoscope for sputum suction	Conventional dose (nebulized 60 mg/d for 7 days) combined with vibration expectoration instrument
Feng 2015	44	44	88	73.3 ± 9.1	72.4 ± 9.7	High-dose ambroxol hydrochloride (nebulized 120 mg/d for 4 days) combined with fiberoptic bronchoscope for sputum suction	Conventional dose (nebulized 60 mg/d for 4 days) combined with vibration expectoration instrument
Li 2013	72	70	142	60–86	60–86	High-dose ambroxol hydrochloride (nebulized 1005 mg/d for 3 days) combined with fiberoptic bronchoscope for sputum suction	Conventional dose (nebulized 90 mg/d for 3 days) combined with vibration expectoration instrument
Han 2017	43	43	86	67.87 ± 5.93	70.11 ± 5.23	High-dose ambroxol hydrochloride (nebulized 300 mg/d for 5 days) combined with fiberoptic bronchoscope for sputum suction	Conventional dose (nebulized 60 mg/d for 5 days) combined with vibration expectoration instrument
Biao 2015	30	30	60	[0,5-6]62.5 ± 5.6	High-dose ambroxol hydrochloride (nebulized 600 mg/d for 3 days) combined with fiberoptic bronchoscope for sputum suction	Conventional dose (nebulized 90 mg/d for 3 days) combined with vibration expectoration instrument
Hao 2015	49	49	98	[0,5-6]6.2 ± 8.7	High-dose ambroxol hydrochloride (nebulized 1005 mg/d for 5 days) combined with fiberoptic bronchoscope for sputum suction	Conventional dose (nebulized 90 mg/d for 5 days) combined with vibration expectoration instrument

RCT = randomized controlled trial.

### 3.2 Quality of included RCTs

**Figure 2:**
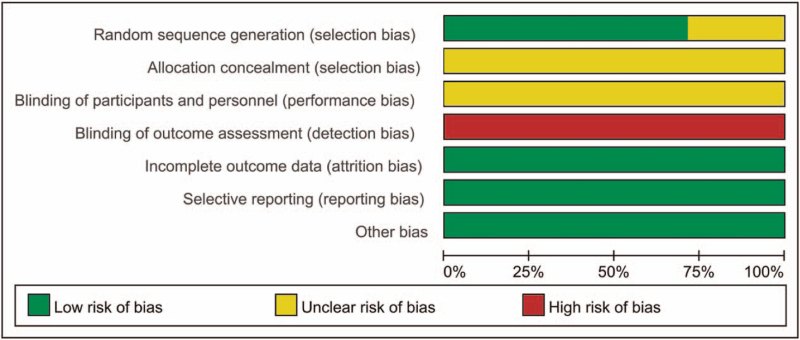
Risk of bias graph.

**Figure 3:**
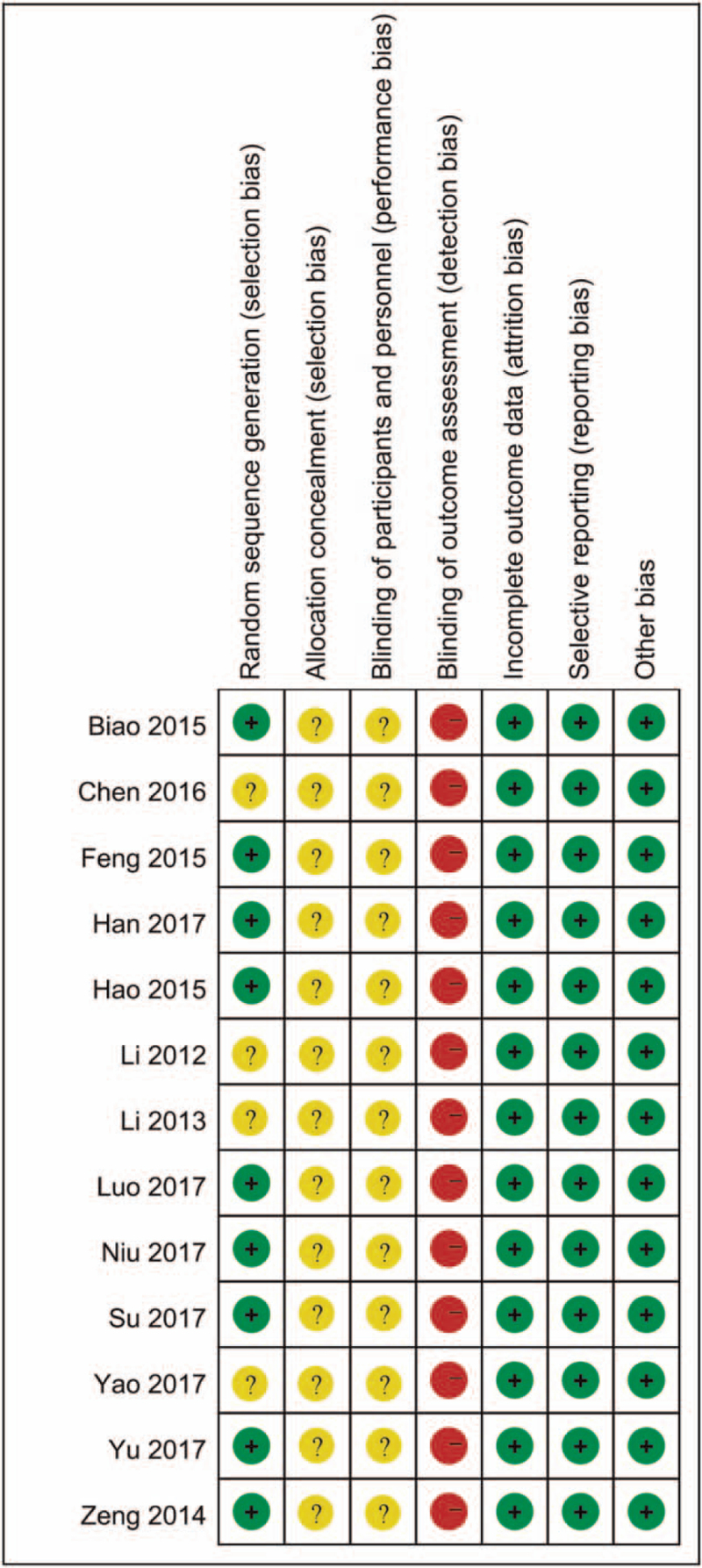
Risk of bias summary.

The quality of the included studies is presented in Figures 2 and 3. Following strict judgments of each included RCT according to the Cochrane handbook, although all of the included RCTs mentioned randomization, only 10 RCTs^[18,20–25,28,29]^ explained the specific details on how to generate the random sequences, 4 RCTs^[17,26,27,30]^ did not provide a detailed description of the methods used to produce a random sequence. All included RCTs did not report allocation blinding or the personnel blinding. For the blinding of outcome assessment, it seems to be difficult to set blinding design to the outcome assessors given the nature of intervention, thereby all the RCTs were all rated as high risk of biases in this domain. No selective reporting or other significant biases amongst the 13 included RCTs were found.

### 3.3 Meta-analyses

#### 3.3.1 Blood oxygen partial pressure

**Figure 4:**
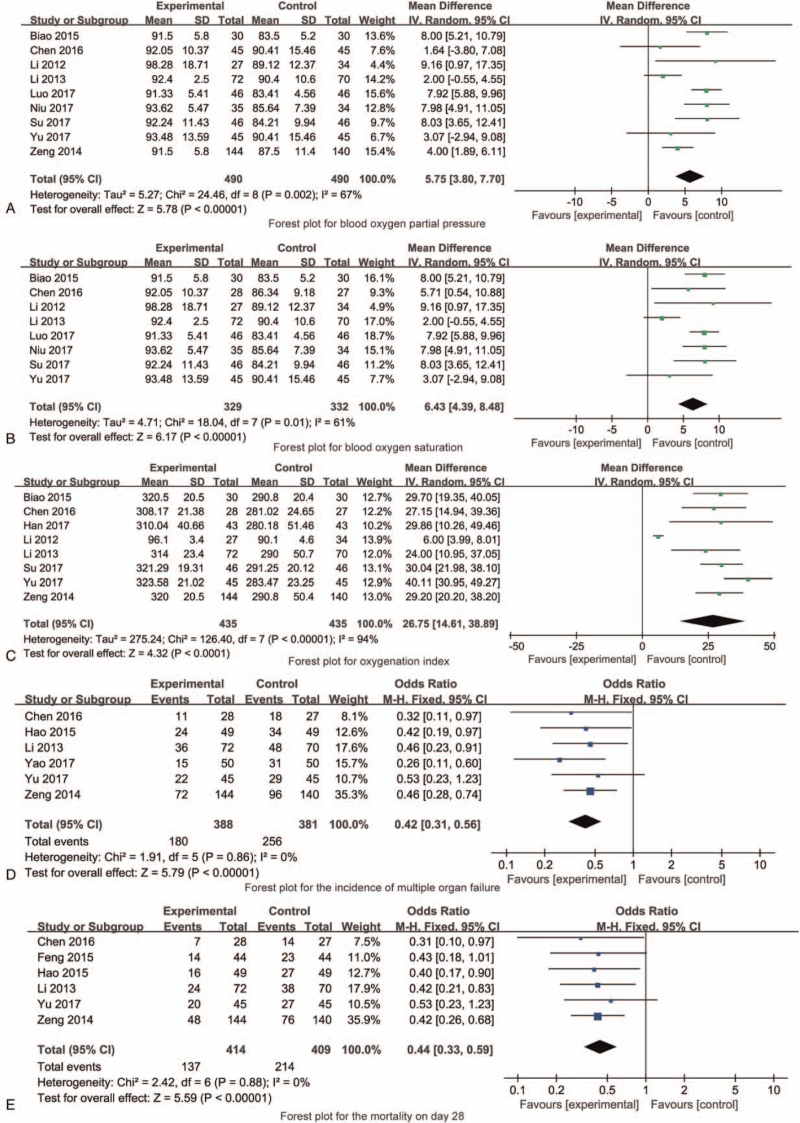
The forest plots for synthesized outcomes.

A total of 9 RCTs reported the blood oxygen partial pressure, the results showed that the blood oxygen partial pressure of experimental group was significantly higher than that of control group [MD = 5.75, 95% CI (3.80, 7.70), *P* < .001, Fig. 4A].

#### 3.3.2 Blood oxygen saturation

A total of 8 RCTs reported the blood oxygen saturation, the results showed that the blood oxygen saturation of experimental group was significantly higher than that of control group [MD = 6.43, 95% CI (4.39, 8.48), *P* < .001, Fig. 4B].

#### 3.3.3 Oxygenation index

A total of 8 RCTs reported the oxygenation index, the results showed that the oxygenation index of experimental group was significantly higher than that of control group [MD = 26.75, 95% CI (14.61, 38.89), *P* < .001, Fig. 4C].

#### 3.3.4 The incidence of multiple organ failure

A total of 6 RCTs reported the incidence of multiple organ failure, the results showed that the incidence of multiple organ failure of experimental group was significantly less than that of control group [OR = 0.42, 95% CI (0.31, 0.56), *P* < .001, Fig. 4D].

#### 3.3.5 The mortality on day 28

A total of 6 RCTs reported the mortality on day 28, the results showed that the mortality until day 28 of experimental group was significantly less than that of control group [OR = 0.44, 95% CI (0.33, 0.59), *P* < .001, Fig. 4E].

### 3.4 Sensitivity analysis and publication bias

**Figure 5:**
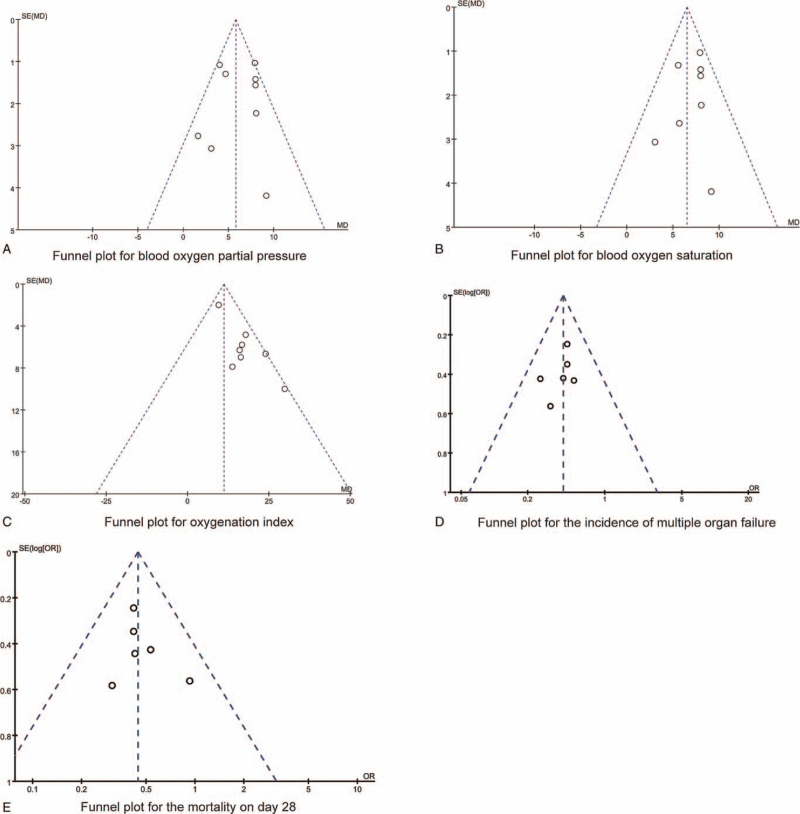
The funnel plots for synthesized outcomes.

Sensitivity analyses were conducted with fixed effects model and random effects model for comparative analysis, the results suggested that the results of each analysis were basically robust and reliable. We used funnel chart to qualitatively identify publication bias. As shown in Figure 5, the graph showed that the distribution of the scattered points of each result index was basically symmetrical, and no publication bias was found by the Egger test (all *P* > .05).

## 4 Discussions

At present, due to the high mortality of severe pneumonia in the elderly, it has become one of the common diseases that endanger the health of the elderly.^[31]^ In recent years, ambroxol hydrochloride has been widely used in the clinical treatment of severe pneumonia with its good anti-inflammatory effect and pulmonary expectorant effect.^[32]^ Studies^[33,34]^ have shown that high-dose ambroxol hydrochloride has a good protective effect on the lungs. However, ambroxol hydrochloride has poor sputum expectoration ability, and it is necessary to combine ambroxol hydrochloride treatment with vibratory expectoration instrument therapy to improve expectoration. However, the vibratory expectoration device has poor ability to clear sputum deep in the respiratory tract, resulting in residual sputum, which can easily cause secondary infections.^[7]^ Fiberoptic bronchoscopy can effectively clear tracheal secretions under visual conditions, and can detect trachea and lung lesions in time, and has become one of the preferred treatments for severe pneumonia.^[35,36]^ Based on the results of the meta-analysis, we have found that the treatment effect of high-dose ambroxol hydrochloride combined with bronchoscopy is superior to the normal-dose ambroxol hydrochloride therapy combined with sputum expectoration.

Ambroxol hydrochloride is a kind of surface active substance activator and mucolytic agent.^[37]^ It can not only dissolve mucus sputum and lubricate the respiratory tract, promote sputum discharge, but also promotes the synthesis and secretion of surface active substances.^[38]^ Some authors^[39,40]^ hypothesize that fiberoptic bronchoscopy can directly infuse the lung segment or lung sub-segment level of the diseased part, clear the airway and alveolar retained substances, and relieve airway obstruction. The fiberoptic bronchoscopy alveolar lavage brush directly reaches the site of the lesion, which can completely remove the sputum in the alveoli.^[41]^ Besides, it can clear the inflammatory secretions in the bronchus, use normal saline to wash repeatedly, promote sputum discharge, relieve airway blockage, effectively improve the hypoxia state of elderly patients with severe pneumonia, and improve the efficacy.^[42]^

Arterial blood gas indicators directly reflect the body's acid-base balance and the degree of hypoxia, and are related to the severity of illness in elderly patients with severe pneumonia.^[43]^ The oxygenation index is mainly used to assess the oxygenation state of the body, and if it is significantly reduced, it indicates lung dysfunction.^[44]^ The results of this study showed that the levels of blood oxygen partial pressure, blood oxygen saturation, oxygenation index of the experimental group after treatment were significantly higher than those of the control group, indicating that the combined treatment would directly perfuse normal saline into the lesion, it clears the deep sputum of the alveoli, reduces the blockage of the respiratory tract, increases the flow of inhaled oxygen, and improves the ventilation function.^[45]^ The results of our findings are consistent with several previous studies.^[36,46]^

This study has certain limitations that must be concerned. Firstly, due to the particularity of clinical trials and ethical requirements, blinding was not used in most included RCTs. Secondly, the search language in this present meta-analysis is limited to Chinese and English, most of the included RCTs that met the inclusion criteria are studies from China. Thirdly, pulmonary infection is a common yet serious complication in COVID19 patients, we conducted updated search for the relevant articles on pulmonary infection specifically COVID19 patients, yet no RCTs on the use of high-dose ambroxol hydrochloride combined with fiberoptic bronchoscopy have been found. Finally, if pursued therapeutic intervention modulates survival outcomes, data must be presented with survival curves. Due to limited data, we could not perform survival curves analysis in this meta-analysis. Therefore, it is still necessary to design larger sample of RCTs for verification in the future to further evaluate the effect of using high-dose ambroxol hydrochloride combined with bronchoscopy in the treatment of severe pneumonia.

## 5 Conclusions

In conclusion, current evidence shows that high-dose ambroxol hydrochloride combined with fiberoptic bronchoscopy is beneficial to improve the prognosis of elderly patients with severe pneumonia. It may be helpful to improve arterial blood gas indicators and lung function with less adverse reactions. However, its role in reducing the mortality of patients still needs to be further verified by prospective clinical studies with larger sample sizes in the future.

## Author contributions

HT, ZY designed research; HT, ZY, JL, QW, WF conducted research; ZY analyzed data; HT, ZY wrote the first draft of manuscript; ZY had primary responsibility for final content. All authors read and approved the final manuscript.

**Conceptualization:** Zhi Yuan, Qun Wang.

**Data curation:** Zhi Yuan, Qun Wang, Weijie Fan.

**Formal analysis:** Haowei Tang, Zhi Yuan, Weijie Fan.

**Investigation:** Zhi Yuan, Weijie Fan.

**Methodology:** Haowei Tang, JingJie Li, Qun Wang.

**Resources:** Haowei Tang, Zhi Yuan.

**Software:** JingJie Li, Qun Wang.

**Supervision:** Haowei Tang, JingJie Li.

**Validation:** Haowei Tang, Zhi Yuan.

**Visualization:** Zhi Yuan.

**Writing – original draft:** Zhi Yuan.

**Writing – review & editing:** Zhi Yuan.
